# Effect of vulcanization temperature and dental stone colour on colour degradation of maxillofacial silicone elastomers

**DOI:** 10.1186/s12903-017-0365-6

**Published:** 2017-03-31

**Authors:** Ebru Demet Cifter, Meltem Ozdemir – Karatas, Emrah Baca, Adem Cinarli, Ali Balik, Erkan Sancakli, Bilge Gokcen-Rohlig

**Affiliations:** 1 Department of Prosthodontics, Faculty of Dentistry, Istanbul University, 34093 Capa-Istanbul, Turkey; 2 Department of Chemistry, Faculty of Engineering, Istanbul University, 34320 Avcilar-Istanbul, Turkey

**Keywords:** Maxillofacial silicone elastomer, Spectrophotometric analysis, Colour change, Dental stone, Temperature, CIEDE2000

## Abstract

**Background:**

Colour degradation is a major problem in maxillofacial silicone elastomers. Recent studies have focused on colour stability and the mechanical properties of the silicone elastomers. A colour match is also essential for the acceptance of the prosthesis by the patient. The aim of this study is to assess the colour degradation of the silicone elastomer after being moulded in different colours of dental stones at two different vulcanization temperatures.

**Methods:**

Five different colours of dental stones were used to fabricate a total of 120 silicone blocks using a Cosmesil M511 maxillofacial silicone elastomer. Vulcanization was completed at two different temperatures (25 and 100° Celsius). Colour measurements were obtained with a Conica Minolta spectrophotometer. The CIEDE2000 formula was used to calculate the colour differences (∆E00). Two-way ANOVA, one-way ANOVA with Bonferroni corrected post-hoc *p* values and independent samples *t*-test were used for the statistical analyses.

**Results:**

High temperature vulcanization causes lightening of the maxillofacial silicone elastomers without regard to the dental stone colour (*p* = 0.001). Specimens moulded in green stone lightened least at room temperature (*p* = 0.999). Compared to the control group, at high temperature, all specimens moulded in coloured dental stones darkened significantly (*p* < 0.001 for white, blue and yellow; *p* = 0.006 for green; *p* = 0.045 for reddish-brown). In the high temperature group, the shift to a green chroma was significant in the white, yellow and green dental stones groups (*p* = 0.001, *p* = 0.002, *p* < 0.001, respectively). The mean b* of the high temperature control group was higher than that of the room temperature control group (*p* < 0.001). The only ∆E00 score lower than the perceptibility threshold for dental materials (∆E00 = 1.30) was between the room temperature control group and the room temperature green dental stone group (∆E00 = 0.96).

**Conclusions:**

Green and blue dental stones cause less colour degradation in silicone elastomers. Reddish-brown dental stones cause the most colour degradation in silicone elastomers. At 100 °C, the colour of the silicone elastomer lightens and yellows even if the elastomer is vulcanized in a stainless steel mould. White, yellow and reddish-brown dental stones make the silicone elastomer appear more yellow even if the elastomer is vulcanized at room temperature.

## Background

Maxillofacial silicone elastomers have been widely used to fabricate facial prostheses for restoring the normal appearance of patients with maxillofacial defects. Silicone elastomers are one of the leading materials available for the fabrication of maxillofacial prostheses. The colour match is the most challenging part of maxillofacial prosthesis fabrication for prosthodontists, and the acceptance of the prosthesis by the patient usually depends on an accurate colour match between the prosthesis and the skin [1, 2]. Although silicone elastomers exhibit acceptable mechanical properties and satisfactory aesthetic results, they should be renewed every couple of years due to colour degradation caused by outdoor weathering, disinfectant use, and staining of the prosthesis due to daily habits [3–9].

The colour changes of dental materials such as light-cured composite resins, resin cements and acrylic polymers used for ocular prostheses during the polymerization process have been presented in various studies [10–15]. However, the colour change of maxillofacial silicone elastomers after vulcanization has not been investigated. The effect of temperature on the lengths of polymer chains or the negative effects of temperature on pigments and dyes used to colour maxillofacial silicone elastomers might be possible factors responsible for the colour change [16–19].

Recent studies have aimed to improve the colour stability and aesthetics of the materials used for the fabrication of maxillofacial prostheses. These studies have focused on the effect of the incorporation of nano-oxides, ultraviolet light absorbers and opacifiers on the colour stability and mechanical properties of silicone elastomers [20–27]. Chu and Fisher [28] reported that adding 1.5% UV absorbers by weight provided adequate UV protection, which is supported by other studies [21, 24, 29]. However, colour stability remains a major problem for maxillofacial patients.

The fabrication of a maxillofacial prosthesis consists of various stages, including taking the impression, construction of a wax sculpture and colour match followed by moulding. Obtaining a detailed impression, sculpting a correctly positioned wax model and developing a proper surface texture are important as are colour selection and processing. Although there is no guidance for dental stone selection in the literature, the master impression of the defect site is usually poured with improved dental stones available in different colours [1]. During moulding, a slight to moderate colour change occurs in the silicone elastomer that is usually visually detectable after the vulcanization is complete. Various factors, such as pigment percentage, ageing and environmental factors are extensively discussed in other studies [3–9], but colour change after moulding has not been mentioned in the dental literature. Colour degradation may be due to colour invasion from the dental stone to the silicone elastomer or vulcanization temperature, and it can be avoided, to some extent, with the use of a separating medium [30]. Nevertheless, using a medium may cause a loss of surface details of the wax model, such as pores and wrinkles, which play an important role in a natural appearance.

The colour parameters of the samples were recorded using Commission Internationale de l’Eclairage (CIE) L*a*b* coordinate system (Commission Internationale de l’Eclairage 1971) which is a uniform colour space where L*a*b* coordinates provide a numerical description of the colour position in a 3-dimensional space. The L* coordinate represents the lightness/darkness in a range from 0 to 100. The a* coordinate represents the greenness/redness of the specimen in a range from -90 to 70. The b* coordinate represents yellowness/blueness in a range from -80 to 100. CIEDE2000, the latest developed formula of CIE, is reported to be more effective in reflecting the colour differences perceived by the human eye [31]. Colour matches for dental materials are evaluated according to the perceptibility and acceptability thresholds A 50:50% perceptibility threshold means that the colour difference between two samples can be detected by 50% of the observers. A 50:50% acceptability threshold means that 50% of the observers find the colour difference acceptable between two samples [32]. Ghinea R et al. used CIEDE2000 and reported the perceptibility threshold as ∆E00 = 1.30 and the acceptability threshold as ∆E00 = 2.25 in dental ceramics [33].

The aim of the current study is to assess the colour degradation of the silicone elastomer after being moulded in different colours of dental stones. Furthermore, the authors hypothesize that this colour change will be exacerbated by vulcanization temperatures. The effects of dental stone colour and vulcanization temperature on the colour of maxillofacial silicon elastomers were evaluated with a spectrophotometer (CM 3600 D, Conica Minolta, Tokyo, Japan).

## Methods

Moulds were prepared using 37 mm x 16 mm x 10 mm dimensioned erasers (Pritt, Henkel, New Zealand) in 5 different commercially available colours of dental stones (Green: Glastone 3000/Dentsply Inc. UK, Reddish-brown: Cam-stone N/Ernst-Hinrichs Dental GmbH-Germany, White/Blue/Yellow: Amberok/Anadolu Dental Products-Turkey). The upper and lower sides of the moulds were isolated with a colourless petrolatum liquid (Visco-gel Dentsply Inc. UK), avoiding touching the eraser surfaces to maintain specimen gaps free of separating medium. After the setting of the dental stone was completed, the erasers were removed from the moulds without the need for a boiling stage. A stainless steel mould was fabricated to obtain control group specimens with the same dimensions as the investigated group specimens. Each group included 10 specimen gaps. Sixty specimens each were prepared for the room temperature and high-temperature groups.

All of the 120 silicone blocks were fabricated using a 10:1 ratio of part A to part B of M511 Maxillofacial Silicone Elastomer (Technovent Ltd, UK). A total of 850 g part A, 85 g Part B and 1.5 g of intrinsic colourant mixture (P105, P108, P112, P410, P413, P414, and P415) were weighed on a digital weighing scale (Acculab Econ, Sartorius AG, Germany) and mixed on a glass slab to obtain an average fair shade Turkish skin colour (Fitzpatrick scale III). The intrinsic colourant weight was less than 0.2% of the silicone elastomer weight to avoid altering the physical and mechanical properties of the elastomer as mentioned in the literature [34].

A coloured silicone elastomer was placed in the stone and stainless steel moulds. Samples in the room temperature vulcanization group were clamped and left at room temperature (25 °C) for 24 h. Samples in the high temperature vulcanization group were clamped and cured at 100 °C for 1 h. The vulcanized silicone samples were trimmed and washed with an air/water spray for 20 s and dried for 30 s.

Colour measurements were made with a spectrophotometer (CM 3600 D, Conica Minolta, Tokyo, Japan) using the Commission Internationale de l’Eclairage (CIE) colour system. The CIE system is a device-independent system, which defines colour with coordinates L* (lightness/darkness), a* (redness/greenness) and b* (yellowness/blueness). The instrument was calibrated with a standard white card (L* = 93.3, a* = 0.9, b* = 2.7) as suggested by the manufacturer before the readings. The same white card was used as a background during the colour reading process to standardize the measurements. Due to its translucent nature, maxillofacial colour readings of silicone elastomers are performed using a background card, as advised in various studies [23, 24, 35–37]. Readings were obtained from 3 adjacent points of the downward looking surface of each specimen, which was in touch with the first poured lower side of the moulds. The L*a*b* data of each specimen were entered on a spreadsheet (Excel 2010, Microsoft). The mean L*a*b* values of 30 measurements, which were obtained from 10 specimens in each group, were recorded as the group’s mean L*a*b* score. ∆E00 was calculated between the room temperature and the high temperature groups as well as between each coloured dental stone sample and its own control group. The colour change (∆E00) was calculated according to the following equation [38].

$$ \varDelta {\mathrm{E}}_{00} = {\left[{\left(\varDelta \mathrm{L}\prime /{\mathrm{k}}_{\mathrm{L}}{\mathrm{S}}_{\mathrm{L}}\right)}^2 + {\left(\varDelta \mathrm{C}\prime /{\mathrm{k}}_{\mathrm{C}}{\mathrm{S}}_{\mathrm{C}}\right)}^2 + {\left(\varDelta \mathrm{H}\prime /{\mathrm{k}}_{\mathrm{H}}{\mathrm{S}}_{\mathrm{H}}\right)}^2 + {\mathrm{R}}_{\mathrm{T}}\left(\varDelta \mathrm{C}\prime /{\mathrm{k}}_{\mathrm{C}}{\mathrm{S}}_{\mathrm{C}}\right)\left(\varDelta \mathrm{H}\prime /{\mathrm{k}}_{\mathrm{H}}{\mathrm{S}}_{\mathrm{H}}\right)\right]}^{0.5} $$

The variables ΔL′, ΔC′ and ΔH′ refer to differences in lightness, chroma and hue between two measurements, respectively. kL, kC, and kH are correction terms for variation in experimental conditions. SL, SC, and SH are weighting functions used to adjust the total colour difference. RT is the rotation function that is related to the interaction between chroma and hue differences in the blue region.

NCSS (Number Cruncher Statistical System) 2007 (Kaysville, Utah, USA) was used for statistical analysis. The data are reported as the mean ± standard deviation. Two-way analysis of variance was conducted to analyse the effects of temperature and dental stone colour on L*, a*, and b* values of maxillofacial silicone elastomers. One-way ANOVA with Bonferroni corrected post-hoc *p* values and independent samples t-tests were conducted to analyse the effects of dental stone colour and temperature differences on L*a*b* values. Statistical significance was set as *p* < 0.05.

## Results

Table 1 shows the mean and standard deviation for L* values with respect to the polymerization temperature and dental stone colour. A positive change in L* indicates lightening of the specimen colour whereas a negative change indicates darkening.

**Table 1:** Differences in mean L* among study groups

Groups	Room temperature	High temperature	^a^ *p*
	Mean L* ± SD	Mean L* ± SD	
Control group	60.56 ± 3.58	64.68 ± 5.21	0.001*
White	65.03 ± 4.15	54.58 ± 4.61	<0.001*
Blue	62.54 ± 4.20	57.36 ± 5.52	<0.001*
Yellow	64.05 ± 4.88	58.74 ± 6.21	0.001*
Green	61.49 ± 4.91	59.71 ± 6.35	0.230
Reddish-brown	62.18 ± 4.34	60.52 ± 3.79	0.119

^a^Independent samples *t* test

*p < 0.05

The 2-factor ANOVA results indicate that the effects of temperature and stone colour and the interaction of temperature and stone colour on ∆L* were significant (*p* < 0.001, *p* = .017, *p* < 0.001, respectively).

The mean L* value of the high temperature control group was higher than the mean L* value of the room temperature control group (*p* = 0.001), indicating that without regard to dental stone colour, an increase in temperature causes lightening of the maxillofacial silicone elastomers (Table 1). With respect to room temperature coloured dental stone specimens, all high-temperature coloured dental stone specimens darkened (L* value decreased) when heated. The decreases in L* were significant in the white, blue and yellow stone groups (*p* < 0.001, *p* < 0.001, and *p* = 0.001, respectively).

Post hoc results (Table 2) showed that, compared to control group, at room temperature, all specimens lightened (L* value increased) when moulded in coloured dental stones. At room temperature the increase in L* in white (*p* = 0.002) and yellow (*p* = 0.035) stone specimens was significant compared to the control group. Specimens moulded in green stone lightened least compared to control specimens (∆L* = 0.93) (*p* = 0.999).

**Table 2:** Post-hoc mean L* comparisons among study groups

Groups	Room temperature		High temperature	
	∆L*	^a^ *p*	∆L*	^a^ *p*
Control/white	−4.47	0.002*	10.10	<0.001*
Control/blue	−1.98	0.999	7.33	<0.001*
Control/yellow	−3.48	0.035*	5.95	<0.001*
Control/green	−0.93	0.999	4.97	0.006*
Control/reddish-brown	−1.62	0.999	4.16	0.045*
White/blue	2.49	0.428	−2.77	0.697
White/yellow	0.98	0.999	−4.15	0.046*
White/green	3.54	0.030*	−5.13	0.004*
White/reddish-brown	2.84	0.188	−5.94	<0.001*
Blue/yellow	−1.51	0.999	−1.38	0.999
Blue/green	1.05	0.999	−2.36	0.999
Blue/reddish-brown	0.35	0.999	−3.16	0.350
Yellow/green	2.56	0.370	−0.98	0.999
Yellow/reddish-brown	1.86	0.999	−1.78	0.999
Green/reddish-brown	−0.69	0.999	−0.81	0.999

^a^Independent samples *t* test

*p < 0.05

Post-hoc results (Table 2) indicate that compared to the control group, at a high temperature, all of the specimens that were moulded in coloured dental stones darkened (L* value decreased) significantly (*p* < 0.001 for white blue and yellow; *p* = 0.006 for green; *p* = 0.045 for reddish-brown).

At high temperature, specimens moulded in white dental stone were darker than specimens moulded in yellow, green and reddish-brown dental stones at the end of the vulcanization (*p* = 0.046, *p* = 0.004 and *p* < 0.001, respectively).

Table 3 shows the mean and standard deviations for a* values (red/green chroma) with respect to polymerization temperature and dental stone colour. A positive change in a* indicates redness of the specimen whereas a negative change indicates greenness.

**Table 3:** Differences in mean a* among study groups

Groups	Room temperature	High temperature	^a^ *p*
	Mean a* ± SD	Mean a* ± SD	
Control group	2.15 ± 0.31	2.30 ± 0.41	0.118
White	2.41 ± 0.42	1.77 ± 0.41	<0.001*
Blue	2.33 ± 0.31	1.96 ± 0.46	<0.001*
Yellow	2.09 ± 0.44	1.81 ± 0.49	0.027*
Green	2.23 ± 0.37	1.29 ± 0.34	<0.001*
Reddish-brown	3.83 ± 0.64	3.56 ± 0.70	0.123

^a^Independent samples *t* test

*p < 0.05

The 2-factor ANOVA results indicate that the effects of temperature and stone colour and the interaction of temperature and stone colour on ∆a* were significant (*p* < 0.001, *p* < 0.001, *p* < 0.001, respectively).

The difference in mean a* between the room temperature control group and the high temperature control group was not significant (*p* = 0.118), indicating that, regardless of the dental stone colour, an increase in temperature did not significantly change the a* values (redness/greenness) of the specimens (Table 3). When coloured stone moulds were heated, the mean a* values of the specimens decreased, indicating that there was a shift in the chroma towards green (Table 3). Decreases in a* values were significant in the white, blue, yellow and green stone groups (*p* < 0.001, *p* < 0.001, *p* = 0.027, *p* < 0.001, respectively).

Post hoc results (Table 4) indicate that in the high temperature group, white, yellow and green dental stone specimens showed significant decreases in mean a* (colour shift towards green) compared to the control group (*p* = 0.001, *p* = 0.002, and *p* < 0.001, respectively). When vulcanization was completed at high temperature, the mean a* values of the green stone specimens decreased significantly (colour shifted towards green) compared to the white, blue, yellow, and reddish-brown specimens (*p* = 0.002, *p* < 0.001, *p* = 0.001, and *p* < 0.001, respectively). When moulded in reddish-brown stone, the mean a* values of the specimens increased significantly (*p* < 0.001) both in the room temperature and high-temperature groups (colour shifted towards red).

**Table 4:** Post-hoc mean a* comparisons among study groups

Groups	Room temperature		High temperature	
	∆a*	^a^ *p*	∆a*	^a^ *p*
Control/white	−0.26	0.287	0.52	0.001*
Control/blue	−0.19	0.999	0.34	0.114
Control/yellow	0.06	0.999	0.48	0.002*
Control/green	−0.09	0.999	1.00	<0.001*
Control/reddish-brown	−1.68	<0.001*	−1.26	<0.001*
White/blue	0.07	0.999	−0.18	0.999
White/yellow	0.32	0.062	−0.04	0.999
White/green	0.18	0.999	0.48	0.002*
White/reddish-brown	−1.42	<0.001*	−1.78	<0.001*
Blue/yellow	0.25	0.394	0.15	0.999
Blue/green	0.10	0.999	0.66	<0.001*
Blue/reddish-brown	−1.49	<0.001*	−1.60	<0.001*
Yellow/green	−0.15	0.999	0.52	0.001*
Yellow/reddish-brown	−1.74	<0.001*	−1.74	<0.001*
Green/reddish-brown	−1.59	<0.001*	−2.26	<0.001*

^a^Independent samples *t* test

*p < 0.05

Table 5 shows the mean and standard deviations for b* values (blue/yellow chroma) with respect to the polymerization temperature and dental stone colour. A positive change in b* indicates yellowness of the specimen whereas a negative change indicates blueness.

**Table 5:** Differences in mean b* among study groups

Groups	Room temperature	High temperature	^a^ *p*
	Mean b* ± SD	Mean b* ± SD	
Control group	3.35 ± 0.49	4.21 ± 0.73	<0.001*
White	4.15 ± 0.78	4.29 ± 0.35	0.380
Blue	3.94 ± 0.55	3.69 ± 0.61	0.100
Yellow	5.77 ± 1.14	5.67 ± 0.90	0.683
Green	3.96 ± 0.52	4.32 ± 0.46	0.006*
Reddish-brown	11.53 ± 1.50	13.77 ± 1.64	<0.001*

^a^Independent samples *t* test

*p < 0.05

The results of the 2-factor ANOVA showed that the effects of temperature, stone colour and the interaction of temperature and stone colour on ∆a* were significant (*p* < 0.001, *p* < 0.001, and *p* < 0.001, respectively).

Mean b* for the high temperature control group was higher than that of the room temperature control group (*p* < 0.001), indicating that heating the maxillofacial silicone elastomer during vulcanization increases mean b* values and causes yellowness in the silicone elastomer (Table 5). When silicone is moulded in green and reddish-brown dental stones, increasing temperature causes a significant increase in the mean b* of the specimens causing yellowness in the silicone elastomer (*p* = 0.006, *p* < 0.001, respectively).

Post-hoc results on Table 6 show that at room temperature, increase in b* values (colour shift towards yellow) between the control group and the white, yellow and reddish-brown groups was significant (*p* = 0.013, *p* < 0.001, *p* < 0.001, respectively). At high temperatures, the reddish-brown and yellow groups exhibited higher mean b* values (colour shifted towards yellow) compared to the control group (*p* < 0.001). Specimens moulded in reddish-brown dental stone exhibited the highest mean b* values compared to all other specimens (*p* < 0.001) both at room temperature and at a high temperature. Specimens moulded in yellow dental stone showed the second highest b* values compared to all other specimens (*p* < 0.001) both at room temperature and at a high temperature. ∆b* between reddish-brown and yellow specimens was also significant at both temperatures (*p* < 0.001).

**Table 6:** Post-hoc mean b* comparisons among study groups

Groups	Room temperature		High temperature	
	∆b*	^a^ *p*	∆b*	^a^ *p*
Control/white	−0.80	0.013*	−0.08	0.999
Control/blue	−0.59	0.192	0.51	0.394
Control/yellow	−2.42	<0.001*	−1.46	<0.001*
Control/green	−0.61	0.154	−0.11	0.999
Control/reddish-brown	−8.18	<0.001*	−9.57	<0.001*
White/blue	0.21	0.999	0.60	0.152
White/yellow	−1.62	<0.001*	−1.38	<0.001*
White/green	0.19	0.999	−0.03	0.999
White/reddish-brown	−7.38	<0.001*	−9.48	<0.001*
Blue/yellow	−1.83	<0.001*	−1.97	<0.001*
Blue/green	−0.02	0.999	−0.63	0.101
Blue/reddish-brown	−7.59	<0.001*	−10.08	<0.001*
Yellow/green	1.81	<0.001*	1.34	<0.001*
Yellow/reddish-brown	−5.76	<0.001*	−8.11	<0.001*
Green/reddish-brown	−7.57	<0.001*	−9.45	<0.001*

^a^Independent samples *t* test

*p < 0.05

∆E00 values calculated for the room temperature control group versus the room temperature coloured dental stone groups are presented in Table 7. At room temperature, the colour difference between the control group and the green dental stone group was below the perceptible threshold (∆E00 < 1.30). At room temperature, the colour difference between the control group and the blue dental stone group was below the acceptable threshold (∆E00 < 2.25).

**Table 7:** ∆E00 scores between room temperature control group and room temperature coloured dental stone groups

Room temperature control group	Room temperature coloured stone groups	∆E00
Control group	White	3,85
Control group	Blue	**1,78**
Control group	Yellow	3,62
Control group	Green	**0,96**
Control group	Reddish-brown	6,43

Bold characters are ∆E00 values lower than the perceptible (∆E00 = 1.30) or acceptable (∆E00 = 2.25) thresholds

∆E00 values calculated for the high-temperature control group versus the high-temperature coloured groups are presented in Table 8. When the temperature was increased during vulcanization, colour degradation became more visible in specimens moulded in coloured dental stones. The colour difference between the control group and all coloured dental stone groups were higher than the perceptible thresholds (∆E00˃2.25) for high-temperature vulcanized samples.

**Table 8:** ∆E00 scores between high temperature control group and high temperature coloured dental stone groups

High temperature control group	High temperature coloured stone groups	∆E00
Control group	White	8,96
Control group	Blue	6,37
Control group	Yellow	5,31
Control group	Green	4,46
Control group	Reddish-brown	7,76

∆E00 values calculated for room temperature versus high-temperature specimens are presented in Table 9. ∆E00 was 3.57 between the room temperature and high-temperature control group, indicating that when the temperature is increased, even if vulcanized in stainless steel moulds, a visually detectable colour change occurred, exceeding an acceptable threshold (∆E00˃2.25). The ∆E00 score between the room temperature green and the high-temperature green group (∆E00 = 2.07) was below the acceptable threshold (∆E00 = 2.25). The colour difference between the room temperature reddish-brown group and the high-temperature reddish-brown group was ∆E00 = 2.15, which is below the acceptable threshold. However, this difference is due to the excessive colour staining effect of the reddish-brown dental stone (compared to control groups) at both temperatures (∆E00 = 6.43 and ∆E00 = 7.76).

**Table 9:** ∆E00 scores between room temperature and high temperature groups

Room temperature groups	High temperature groups	∆E00
Control group	Control group	3,57
White	White	9,25
Blue	Blue	4,58
Yellow	Yellow	5,31
Green	Green	**2,07**
Reddish-brown	Reddish-brown	**2,15**

Bold characters are ∆E00 values lower than the acceptable (∆E00 = 2.25) thresholds

## Discussion

The current study investigated the colour change of a maxillofacial prosthetic silicone elastomer after vulcanization in five different coloured dental stone moulds at two different temperatures. The null hypothesis was rejected. Both the vulcanization temperature and the dental stone colour affected the colour of the silicone elastomer.

Colour instability is a major problem in maxillofacial silicone elastomers. The tendency of elastomers to turn yellow over time is due to the nature of all silicone elastomers and cannot be avoided. Because of colour degradation, the life span of a maxillofacial prosthesis is limited to 6 to 12 months [20]. Disinfecting procedures, cleansing solutions, the presence of ultraviolet light, and organic pigments are known to adversely affect colour stability [20, 21, 39, 40]. In addition to these factors, in the authors’ experience, maxillofacial silicone elastomers also display a colour change immediately after vulcanization is complete, before the prostheses are exposed to environmental conditions. Because of the behaviour of scattering light, wet surfaces appear darker than dry surfaces [41]. The colour difference just after vulcanization may be due to this effect. However, the results of the present study also show that the increasing temperature during vulcanization causes significant colour changes in the silicone elastomer compared to the room temperature control group. This means that the temperature itself affects the colour of the silicone elastomer. The determination of the effect of dental stone colour and increasing temperature on the silicone elastomer was the main focus of the current research. Five different dental stone colours were chosen in this study. The selected colours were the most readily available dental stone colours in the market and are used in the fabrication stages of the maxillofacial prosthesis. Three different brands of stones were used in the study as the colour options are limited in the dental market. Specimen gaps in the moulds were kept free of any separating medium to determine the direct effects of dental stone pigments on silicone elastomers. The results demonstrated that the stone colour affected the silicone elastomer and led to a statistically significant degradation in every colour. Therefore, colour degradation of a silicone elastomer during the fabrication stages should be taken into consideration when the silicone and dental stone are in contact with each other. According to the results of this study, the selection of a green dental stone as moulding material for the vulcanization stage in the fabrication of maxillofacial prostheses can be advised as this colour demonstrated the lowest degradation at room temperature. This study supports the results of the only previous study on the effect of moulding material on the colour of maxillofacial prostheses [30].

Determination of skin colour and matching the skin colour to the silicone is a challenging procedure for prosthodontists. Differences in colour between the skin and the prosthesis can be detected with colour measuring instruments and during colour degradation. Spectrophotometers are widely used for detecting colour differences of dental materials and are essential for reliable evaluations [42–45]. ∆E00 values are usually used to describe whether the changes in the overall shade are perceptible to the human eye [46]. Ghinea R et al. [33] presented perceptibility and acceptability thresholds for dental ceramics using the CIEDE2000 colour formula. ∆E00 = 1.30 was determined to be the perceptible colour change between two materials, which means that 50% of the observers detected the colour difference. ∆E00 = 2.25 was considered as an acceptable colour difference in the same study. Under perfectly controlled in vitro conditions rather than uncontrolled clinical conditions, small colour differences would be detectable and the perceptible threshold could be much lower. Furthermore, there is a need in the dental literature for comprehensive clinical trials presenting perceptibility and acceptability thresholds for maxillofacial silicone elastomers using the CIEDE2000 colour formula. In the current study, the colour measurements were determined with a CM 3600D spectrophotometer, and 3 measurements were recorded from each specimen to obtain reliable mean L*a*b* values. The mean colour difference, ∆E00, was calculated according to the CIEDE2000 colour formula. The only ∆E00 score below the perceptibility threshold was between the room temperature control group and the room temperature green group (∆E00 = 0.96). Blue dental stone samples showed acceptable colour change at room temperature (∆E00 = 1.78). As the temperature is increased during vulcanization, the green and blue stones caused colour degradation in the silicone samples that exceeded the acceptable thresholds (Table 8). This result supports the hypothesis that increase in temperature during vulcanization causes pigments to penetrate from the stone to the silicone elastomer.

Another aim of this research study was to determine the effect of vulcanization temperature on the colour of silicone elastomers. The silicon elastomer is classified as HTV (High Temperature Vulcanization) and RTV (Room Temperature Vulcanization) by its curing temperature. In the current study, the M511 maxillofacial silicone elastomer was chosen as it is one of the most widely used materials for the prosthetic rehabilitation of facial defects. Manufacturers advise to vulcanize maxillofacial HTV silicone elastomers at 100 °C for 1 h. However, at the end of the vulcanization, the silicone elastomer removed from the moulds has a different colour from the remnant silicone elastomer which is left at room temperature on a glass slab. In our research study, to evaluate the effect of increasing temperature during vulcanization, the study groups were designated as high temperature and room temperature. Both temperatures were investigated in this study, and the results demonstrated statistically significant colour changes when the samples were vulcanized at 100 °C. To the best of our knowledge, there is no other study in the dental literature investigating the effects of temperature on maxillofacial silicone elastomer colour.

As far as the specific coordinates of the CIE are concerned, L*a*b* values were affected both by dental stone colour and temperature. All specimens moulded in coloured dental stones showed a significant decrease in mean L* with increasing heat compared to the high temperature control group. This result may indicate that in the presence of coloured dental stone, the colour pigments penetrate into the silicone elastomer and lead to degradation.

Surprisingly, different stone colours demonstrated different effects on the silicone elastomers in the current study. Reddish-brown stone specimens displayed a colour shift towards red at both temperatures compared with other coloured stone groups and control groups, and the reddish-brown stone specimens had higher a* values compared to all other groups with a *p* < 0.001 significance level. Considering that a major problem with maxillofacial elastomers is yellowing over time, silicone elastomers should not be vulcanized in yellow, reddish-brown or white dental stone moulds, which make the elastomer appear more yellow before the prosthesis is delivered to the patient. Another result obtained in this research study was that silicone elastomers became greener when they were moulded in white, blue, yellow or green stones at 100 °C. Yellow and reddish-brown dental stones resulted in a more yellow appearance of the silicone elastomers at both temperatures. Furthermore, white dental stone specimens appeared more yellow when vulcanized at room temperature. This finding should be kept in mind when silicone elastomers are vulcanized at 100 °C; eventually, an extrinsic colour adjustment will be needed.

The background of dental restorative materials is a combination of tooth substrates and the dark intraoral cavity. However, the most translucent part of the maxillofacial silicone elastomers is at the edges of the prosthesis, where the elastomer overlays the skin. While investigating the masking ability and translucency of porcelain specimens, white and black backgrounds and simulated tooth substrates in different shades can be used as backgrounds [47, 48]. The background colour can make a difference in observations in translucency evaluations. Since this is not a colour matching study, the white background (L* = 93.3, a* = 0.9, b* = 2.7) was used only to standardize the measurements during the colour readings. In the studies which are evaluating the colour match between the skin and the silicone elastomer, a skin-coloured background should be used during the spectrophotometric measurements, since the colour is chosen over the skin of the patient. The possible effects of dark- and fair skin-coloured backgrounds need to be investigated.

One of the limitations of this study was the lack of a non-coloured dental stone group in the study design, which is not available in the dental market. Not all dental stone colours could be obtained from a single manufacturer since they produce limited colour options. The material effect should be evaluated in further studies. A fair skin colour was selected to prepare the specimens involved in this study. The effects of dental stone colour on darker specimens should be investigated in further studies.

The results of this study suggest that silicone elastomers should be cured at room temperature. Future research should address whether RTV silicones or HTV silicones should be used to obtain a satisfactory colour match between the skin and the prosthesis. Additionally, the mechanical properties of HTV silicones vulcanized at room temperature should be investigated.

## Conclusions

Within the limitations of the present study, the following conclusions were drawn:

1. Green dental stone does not cause perceptible colour degradation in the silicone elastomer when vulcanized at room temperature.
2. The colour degradation in the silicone elastomer caused by blue dental stone is below the acceptable thresholds when vulcanized at room temperature.
3. The reddish brown dental stone causes the most colour degradation in the silicone elastomer when vulcanized at 100 °C.
4. At 100 °C, the colour of the silicone elastomer lightens and yellows even if it is vulcanized in a stainless steel mould.
5. At 100 °C, the colour of the silicone elastomer darkens if it is vulcanized in a coloured dental stone mould.
6. White, yellow and reddish-brown dental stones make the silicone elastomer appear more yellow even if they are vulcanized at room temperature.

## Abbreviations

∆E00: Colour difference
°C: Degrees celsius
a*: Coordinates of the CIE system indicating redness or greenness
ANOVA: Analysis of variance
b*: Coordinates of the CIE system indicating yellowness or blueness
CIE: Commission Internationale de l’Eclairage
HTV: High temperature vulcanization
L*: Coordinates of the CIE system indicating lightness or darkness
NCSS: Number Cruncher Statistical System
RTV: Room temperature vulcanization
SD: Standard deviation
